# Identification of candidate mechanosensory transduction channels in the aquatic insect *Protohermes xanthodes* (Megaloptera: Corydalidae) by transcriptome analysis

**DOI:** 10.1093/jisesa/ieaf102

**Published:** 2025-11-22

**Authors:** Yue Zhang, Mengqing Zhang, Zhi Li, Lifang Jiang, Jingyi Chen, Lijun Zheng, Wei Zhao, Xihui Wang, Yechen Tan, Xinglong Huang, Zhengwei Wu

**Affiliations:** Hunan Provincial Key Laboratory of Ecological Conservation and Sustainable Utilization of Wulingshan Resources, School of Life Sciences, Jishou University, Jishou, China; Hunan Provincial Key Laboratory of Ecological Conservation and Sustainable Utilization of Wulingshan Resources, School of Life Sciences, Jishou University, Jishou, China; Hunan Provincial Key Laboratory of Ecological Conservation and Sustainable Utilization of Wulingshan Resources, School of Life Sciences, Jishou University, Jishou, China; Hunan Provincial Key Laboratory of Ecological Conservation and Sustainable Utilization of Wulingshan Resources, School of Life Sciences, Jishou University, Jishou, China; Hunan Provincial Key Laboratory of Ecological Conservation and Sustainable Utilization of Wulingshan Resources, School of Life Sciences, Jishou University, Jishou, China; Hunan Provincial Key Laboratory of Ecological Conservation and Sustainable Utilization of Wulingshan Resources, School of Life Sciences, Jishou University, Jishou, China; Hunan Provincial Key Laboratory of Ecological Conservation and Sustainable Utilization of Wulingshan Resources, School of Life Sciences, Jishou University, Jishou, China; Hunan Provincial Key Laboratory of Ecological Conservation and Sustainable Utilization of Wulingshan Resources, School of Life Sciences, Jishou University, Jishou, China; Hunan Provincial Key Laboratory of Ecological Conservation and Sustainable Utilization of Wulingshan Resources, School of Life Sciences, Jishou University, Jishou, China; Hunan Provincial Key Laboratory of Ecological Conservation and Sustainable Utilization of Wulingshan Resources, School of Life Sciences, Jishou University, Jishou, China; Department of Agronomy, College of Coastal Agricultural Sciences, Guangdong Ocean University, Zhanjiang, China

**Keywords:** Aquatic insect, *Protohermes xanthodes*, Piezo-type mechanosensitive ion channel, no-mechanoreceptor potential C, tissue distribution

## Abstract

*Protohermes* species are among the top aquatic predators in benthic invertebrate communities. Their ability to perceive mechanical stimuli may be functional in mediating responses to freshwater environmental cues. In this study, we performed a transcriptome analysis of the antennae, maxillae, and labium of *Protohermes xanthodes* Navás larvae (Megaloptera: Corydalidae), identifying 2 transmembrane protein genes (*PxanPiezo* and *PxanNompC*), which are phylogenetically related to mechanosensory transduction channels in other insects. PxanPiezo is a large transmembrane protein featuring 38 transmembrane helices that mediate its association with the cytoplasm. PxanNompC is another transmembrane protein containing an extensive intracellular ankyrin repeat domain, a structural feature that enables potential interactions with the cytoskeleton. *PxanPiezo* is widely expressed in the internal tissues, such as nerve tissue, the digestive organs, the excretory organ, and the energy storage tissue, as well as in the body surface tissues, including the antennae, maxillae, labium, legs, lateral filaments, anal prolegs, and the abdominal integument. In contrast, *PxanNompC* exhibits tissue-specific expression in body surface structures and is highly expressed in the abdominal integument. These results suggest that PxanPiezo and PxanNompC possess the structural basis required to convert mechanical stimuli into signal currents. Their distinct structural features and expression profiles imply potential differences in their mechanoelectrical transduction mechanisms. Furthermore, identifying mechanoelectrical transduction channel genes in *P. xanthodes* could facilitate the studies on the sensory mechanisms of this aquatic insect adapting the freshwater environments.

## Introduction

The ability to sense and respond to external and internal mechanical stimuli, such as touch, impact, compression, and tension, is essential for animal survival (Wang et al. 2021, Berni 2024). In insects, mechanosensation relies on mechanoelectrical transduction channels, which are mechano-gated ion channels that are activated by membrane stretch or the tension of force-conveying tethers (Hehlert et al. 2025). The Piezo-type mechanosensitive ion channel (Piezo) and No mechanoreceptor potential C (NompC) are mechanoelectrical transduction channels that are expressed in the internal and surface tissues of insects (Coste et al. 2012, Hehlert et al. 2025). Piezo is a large transmembrane protein comprising 38 transmembrane helices. It exists as trimers on the cell membrane and is functionally involved in the transduction of membrane stretch into ionic currents (Yang et al. 2022). NompC belongs to the transient receptor potential (TRP) family and contains a large intracellular domain composing of plentiful ankyrin repeats. The core of the ankyrin repeat appears to be a helix–loop–helix structure, which is organized into a helical spring associated with cellular tension (Chuang and Chen 2022). The molecular properties of Piezo and NompC in the transduction of membrane stretch and cellular tension enable insects to perceive a wide variety of mechanical stimuli.

Piezo is extensively distributed across both invertebrates and vertebrates, and represents the largest ion channel subunit identified to date (Jin et al. 2020, Min et al. 2021). These channels exhibit a unique structural architecture essential for their mechanotransduction functions (Zhao et al. 2018). Structurally, Piezo channels exist as trimers, adopting a 3-bladed, propeller-like configuration (Wang et al. 2019, Oh et al. 2021). Each blade consists of 9 transmembrane helix units (THUs), each of which is composed of 4 transmembrane helices (Saotome et al. 2018, Yang et al. 2022). The whole blade is highly associated with the cytomembrane and gate the central pore through an anchor domain composed of several semitransmembrane helices (Kefauver et al. 2020, Xiao 2024). This specialized structural architecture enables Piezo channels to function as mechanotransducers in diverse bihavioral and physiological processes. Evidence in the literature has shown that insect Piezo channels are crucial for mechanical sensory such as touch, proprioception, and interoception, and highly associated with insect behavior, organ function and homeostasis (Zhou and Martinac 2023, Xiao 2024). *Bombyx mori* Piezo facilitates sperm maturation by regulating peristaltic squeezing during development (Zhang et al. 2024). In *Drosophila*, Piezo-expressing neurons in male genitalia stabilize copulation posture, while gut-innervating Piezo neurons detect tissue distension to control feeding behavior and prevent overconsumption (Yamanouchi et al. 2023, Zhang et al. 2024). In *Mythimna separata*, females preferentially deposit eggs in the leaf sheath, thereby shielding their eggs from parasitoids; Piezo was confirmed to mediate the oviposition site selection by sensing mechanical cues (Ma et al. 2025). Additionally, Piezo is found in the digestive organs of insects and is involved in regulating defecation and gut motility (Puri et al. 2025). Therefore, Piezo is an important sensory ion channel that integrates mechanical signals into biological responses and has essential roles in insect behaviors and physiological processes.

NompC is primarily found in invertebrates, belonging to the TRP-No mechanoreceptor potential C (TRPN) subfamily of TRP channels, which are divided into 7 subfamilies based on their primary amino acid (Hehlert et al. 2021, Chuang and Chen 2022). The other 6 subfamilies are TRP-Canonical (TRPC), TRP-Ankyrin (TRPA), TRP-Vanilloid (TRPV), TRP-Melastatin (TRPM), TRP-Mucolipin (TRPML), and TRP-Polycystin (TRPP) (Guo et al. 2020, Su et al. 2025a). NompC functions as homotetramers with an intracellular ankyrin repeat domain and a membrane TRP domain (Jin et al. 2017). The ankyrin repeat domain associates with microtubules and acts as a gating spring, transmitting mechanical force to the channel pore (Sun et al. 2019, Zhang et al. 2023). Cryo-electron microscopy (Cryo-EM) studies reveal that ankyrin repeat domain in NompC compresses under force, triggering a clockwise rotation of the TRP domain to open the channel (Wang et al. 2022). Insect NompC is essential for mechanosensation, mediating touch, balance. *Drosophila* NompC contributes to the auditory transduction in the adults, where its continuous turnover and activity-dependent transcription maintain sensory homeostasis (Boyd-Gibbins et al. 2021, Li and Yan 2021). In *Bactrocera dorsalis*, NompC knockout causes lethality, deformed wings/legs, and impaired locomotion, highlighting its role in developmental integrity and behavior (Su et al. 2025b). Additionally, NompC collaborates with Piezo channels in pharyngeal neurons to regulate food swallowing by monitoring cibarium mechanics (Qin et al. 2024). The tether-gating mechanism of NompC enables insects to sense and respond to external and internal mechanical stimuli by detecting cellular compression and shrinkage.

*Protohermes xanthodes* Navás (Corydalidae, Megaloptera) is an aquatic insect of great ecological importance in the freshwater environment (Liu et al. 2013, Wen et al. 2025a). Its larvae are known for their strong mandibles and are among the top aquatic predators in benthic invertebrate communities, preying on Trichopteran and Dipteran insects, as well as other aquatic arthropods (Yang et al. 2025). Therefore, *P. xanthodes* can serve as a valuable bioindicator for assessing the health of freshwater communities and monitoring environmental impacts. Identification of neuronal genes and antioxidant enzymes in this species has provided candidate molecular markers for study the toxic effects of environmental pollutants on aquatic insects (Yang et al. 2024, Yang et al. 2025). Mechanosensation enables insects to perceive various biotic and abiotic mechanical stimuli, which is crucial for aquatic insects to adapt to freshwater environments, by helping them to detect prey and predators, as well as water movement and other stimuli (Hehlert et al. 2025). However, the molecular basis of mechanosensation in *P. xanthodes* larvae still remains unclear. In this study, we conducted transcriptomic analyses of the antennae, maxillae, and labium of *P. xanthodes* larvae. Two candidate mechanoelectrical transduction channels, PxanPiezo and PxanNompC, were identified and characterized further through structural and expression profiling.

## Materials and Methods

### Insects and Pesticide Treatment

The larvae of *P. xanthodes* were collected from Donghe National Wetland Park (28°17′N, 109°28′E) in Hunan Province. After collection in the field, the larvae were reared in a laboratory water environment (Wen et al. 2022). Larvae with a head capsule width of approximately 4 mm were dissected to obtain the following tissues: antennae, maxillae, labium, antennae, thoracic legs, lateral filaments, anal prolegs, and abdominal integument. Three replicates were prepared for each tissue. The tissues intended for RNA-seq analysis and reverse transcription quantitative polymerase chain reaction (RT-qPCR) template preparation were collected and frozen in liquid nitrogen. Each tissue was biologically repeated 3 times.

### RNA-Seq Analysis

Transcriptome sequencing of the maxillae, labium, and antennae was performed on a NextSeq 500 system (Biomarker Technologies, China). Raw reads generated from the cDNA library were stringently filtered by removing reads with a sequencing adaptor, >10% indeterminate bases or >50% bases with a quality score of Q ≤ 20. Unigenes were generated by assembling clean reads with Trinity software (Wen et al. 2025b). Gene annotation was conducted by performing a BLAST search against various databases, including NCBI nonredundant protein sequences (Nr), NCBI nucleotide sequences (Nt), Protein family (Pfam), euKaryotic Ortholog Groups, Swiss-Prot, Kyoto Encyclopedia of Genes and Genomes (KEGG), and Gene Ontology (GO). Fragments per kilobase of transcript sequence per million reads (FPKM) were used to analyze gene expression levels.

### Gene Identification and Sequence Analysis

Candidate Piezo and TRP gene sequences were identified by analyzing the transcriptome presented in this study (GenBank BioProject: PRJNA1310214) and our previous transcriptome data (GenBank BioProject: PRJNA1013730) (Yang et al. 2024). Sequence identity analysis was conducted using the online BLAST service (https://blast.ncbi.nlm.nih.gov/Blast.cgi). The molecular weight (Mw) and the theoretical isoelectric point (pI) were predicted using the Compute pI/Mw web service (Wilkins et al. 1999). Transmembrane helices were predicted using the DeepTMHMM online tool (https://services.healthtech.dtu.dk/services/DeepTMHMM-1.0/). The CD-search tool was applied to identify the conserved domains and the protein family attribution of the putative proteins (Wang et al. 2023). Phylogenetic trees were constructed using the maximum likelihood method in MEGA 11 software (bootstrap method, 1,000 replicates).

### Homology Modelling and Protein Structure Analysis

Three-dimensional protein structures of Piezo and NompC were constructed by homology modelling in SWISS-MODEL (Waterhouse et al. 2018). The protein structure templates were MmusPiezo1 (6LQI_A) in *Mus musculus* and DmelNompC (5vkq.1.A) in *Drosophila* (Jin et al. 2017, Geng et al. 2020). The stereochemical quality of the protein structures was checked using the structure assessment tool of SWISS-MODEL (Waterhouse et al. 2024). The protein structures were visualized by PyMOL software (Schrödinger, United States).

### Gene Expression Analysis

The gene expression of Piezo and TRPs in the head, antennae, maxillae, labium, thorax, foregut, midgut, hindgut, Malpighian tubules, fat body, tracheae, and nerve cord was analyzed using the FPKM method based on the transcriptome data. An FPKM value below 1 was defined as indicating a low expression level of the gene in that tissue. RT-qPCR was conducted in a QuantStudio1 system (ThermoFisher, United States) to investigate the gene expression levels of Piezo and NompC in the antennae, maxillae, labium, thoracic legs, lateral filaments, prolegs, and integument of larvae. RNA was extracted from the tissues by using an RNA extraction kit (Transgen, China). RNA integrity was checked by agarose gel electrophoresis. First-strand cDNA was synthesized via reverse transcription with a first-strand cDNA synthesis kit (Transgen, China). Primers for RT-qPCR were designed based on the gene sequence (Supplementary Table S1). Piezo and NompC genes expression levels in different tissues were calculated using the 2^−ΔΔCt^ method. All data were normalized to α-tubulin expression level (Yang et al. 2024). Gene expression level in prolegs was used for calibrating the relative fold changes. One-way ANOVA was used to analyze the significance of differences in gene expression levels.

## Results

### Transcriptome Overview

**Fig. 1.**
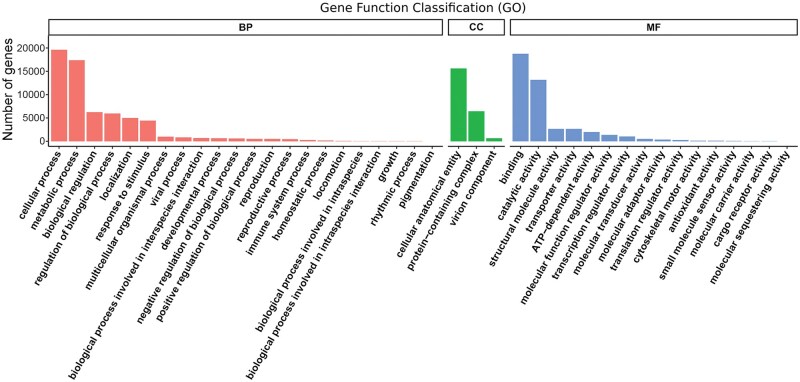
Histogram description of GO enrichment. Genes are assigned to 3 categories: Biological process (BP), Cellular component (CC), and Molecular function (MF). The top 41 most significant GO terms include 26 in BP (Orange bars), 5 in CC (Green bars), and 12 in MF (Blue bars). The *x* axis is the GO term. The *y* axis is the number of genes.

**Fig. 2.**
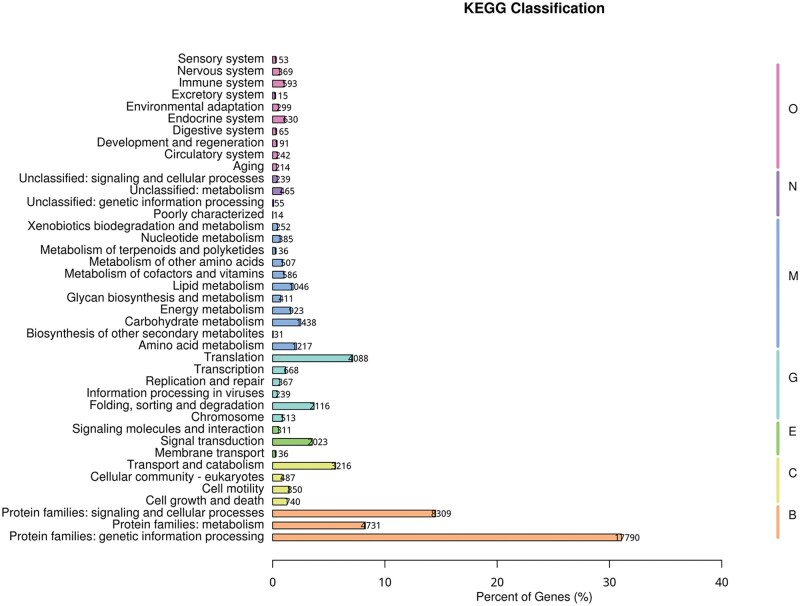
KEGG classification. Genes are assigned to 7 categories: Brite Hierarchies (B), Cellular Processes (C), Environmental Information Processing (E), Genetic Information Processing (H), Metabolism (M), Not Included in Pathway or Brite (N), Organismal Systems (O). The top 41 most significant KEGG terms are shown.

To reveal the sensory adaptation of *P. xanthodes* larvae to the aquatic environment, RNA sequencing of the antennae, maxillae, and labium was performed, presenting 328,944,189 clean reads (Supplementary Table S2). The Q20 of each sample ranged from 98.91 to 99.05, and the GC percentage are 40.23 to 42.21. A total of 177,000 transcripts (median length 609 bp) were obtained by Trinity assembly, producing 101,286 unigenes (median length 530 bp) (Supplementary Table S3). The N50 of the transcripts and unigenes are 2,029 and 1,172, respectively. Gene annotations were conducted by BLASTing against various databases. Of the unigenes, 67.05% (67,921) were annotated in at least 1 database, with the NR database accounting for the highest percentage at 48%. (Supplementary Table S4). In total, 35,683 genes were annotated in the GO database and were assigned to 1,187 functional terms in 3 groups: 828 in “Biological process,” 184 in “Cellular component,” and 175 in “Molecular function” (Fig. 1). In total, 4,449 genes were assigned to “Response to stimulus,” which is one of the most enriched functional terms in “Biological process,” and 30,125 genes were annotated in KO database and mapped to 335 KEGG signal/metabolic pathways (Fig. 2). “Protein families: genetic information processing,” “Protein families: signaling and cellular processes,” “Protein families: metabolism,” “Translation,” and “Transport and catabolism” are among the most enriched pathways.

### Characterization of Piezo in *P. xanthodes*

**Fig. 3.**
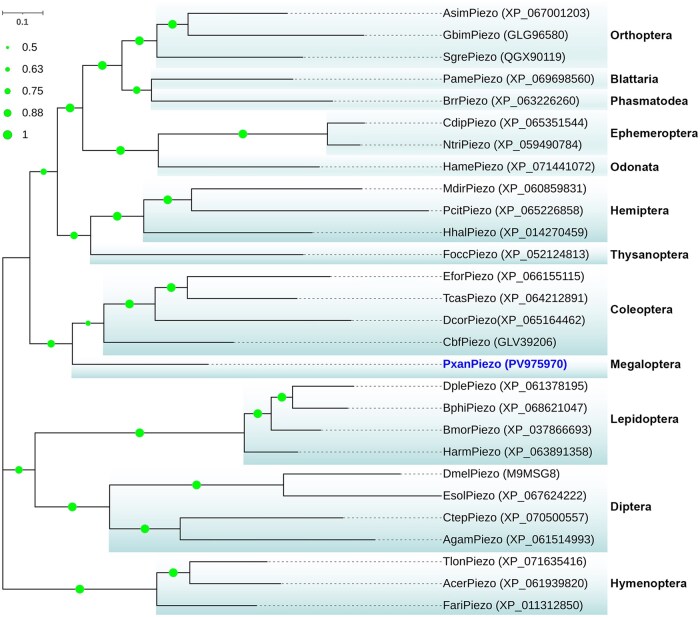
Phylogenetic and structural analysis of PxanPiezo. Insect Piezos cluster into branches that correspond to insect orders. The GenBank accession numbers are given in the brackets after the TRP names. Bootstrap values below 50% are not shown.

The Piezo gene (*PxanPiezo*) identified in *P. xanthodes* larvae contains a 7,470-bp-long open reading frame (ORF), encoding a protein with 2,489 amino acids (Table 1). The predicted Mw of this protein is 286.92 kDa and the theoretical pI is 7.29. BLASTp analysis showed that PxanPiezo contains the conserved domains “PIEZO superfamily” (cl24478) and “Piezo_RRas_bdg” (pfam12166). PxanPiezo shares a close relationship with insect Piezos, and the identities with Piezos in *Schistocerca gregaria*, *Apis cerana*, *B. mori*, *Cloeon dipterum*, and *Drosophila melanogaster* were found to be 50.25%, 46.81%, 45.71%, 43.58%, and 37.13%, respectively. Furthermore, PxanPiezo also exhibits high-sequence similarity with Piezos in noninsect animals, with identities of 39.30%, 32.31%, and 27.40% with Piezos in *M. musculus* (Q8CD54 and 6LQI_A) and *Caenorhabditis elegans* (A0A061ACU2), respectively. In the phylogenetic tree, insect Piezos clusters into branches that correspond to insect orders (Fig. 3). PxanPiezo neighbors the branch of Coleoptera Piezos. It is closely related to Piezos from *Carabus blaptoides fortunei* (GLV39206), *Tribolium castaneum* (XP_064212891), *Euwallacea fornicatus* (XP_066155115) and *Dalotia coriaria* (XP_065164462), with identities of 58.03%, 52.08%, 49.73% and 46.98%, respectively.

**Table 1.** Piezo and TRPs in *P. xanthodes*

Family	Gene	ORF length (bp/aa)	Mw (kDa)	pI	Accession No.
**Piezo**	*PxanPiezo*	7,470/2,489	2,869.21	7.29	PV975970
**TRPA**	*PxanTRPA1*	3,501/1,166	1,324.08	6.10	PX309108
	*PxanPain1*	2,553/850	990.40	7.72	PX309109
	*PxanPain2*	2,538/845	960.43	5.68	PX309110
	*PxanPain3*	2,808/935	1,071.06	5.96	PX309111
	*PxanPain4*	2,832/943	1,058.91	5.18	PX309112
	*PxanWtrw*	2,892/964	1,082.62	6.36	PX309113
	*PxanPyx*	2,562/853	951.72	6.31	PX309114
**TRPC**	*PxanTRP*	3,219/1,072	1,238.05	5.84	PX309115
	*PxanTRPgamma1*	3,816/1271	1,434.06	6.66	PX309116
	*PxanTRPgamma2*	2,430/809	921.52	5.09	PX309117
**TRPM**	*PxanTRPM*	5,019/1,670	1,906.81	5.91	PX309118
**TRPN**	*PxanNompC*	5,238/1,745	1,906.10	7.35	PX309119
**TRPV**	*PxanNan*	2451/816	935.98	5.38	PX309120

**Fig. 4.**
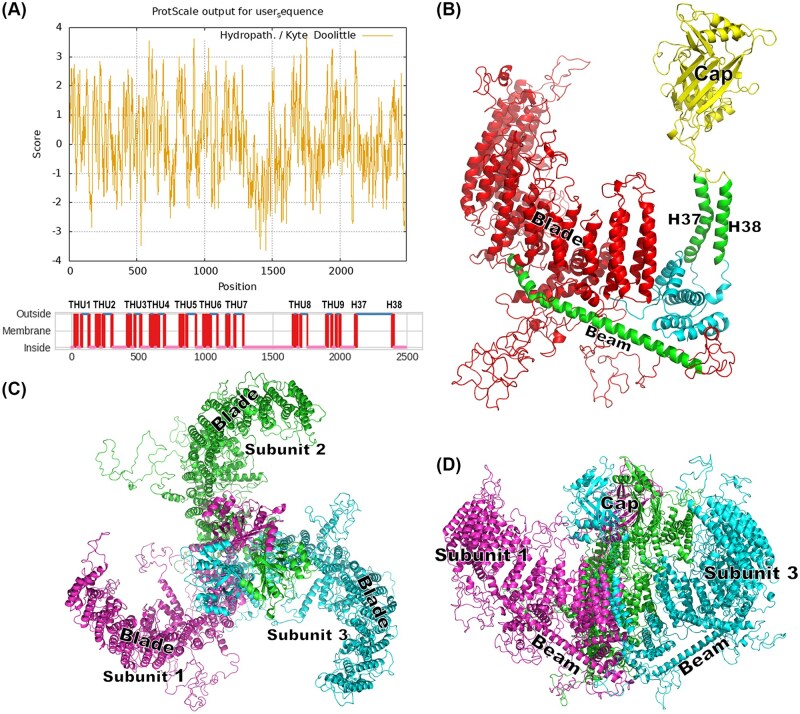
Structural analysis of PxanPiezo. A) Predicted hydrophobic regions and transmembrane helices in PxanPiezo. Positive score indicates hydrophobicity. THU1-9 are transmembrane helix units formed by 36 transmembrane helices. B) Ribbon diagram of the structure of PxanPiezo. Blade structure formed by THUs, beam structure, cap structure were found in PxanPiezo. C) Top view of PxanPiezo trimer. Top view of the trimer is a propeller-shaped structure with 3 blades. D) Side view of PxanPiezo trimer. The side view resembles a funnel covered with a central cap.

Structural analysis showed that PxanPiezo is a large transmembrane protein with intracellular N- and C-terminal ends. It contains 38 transmembrane helices (H1–38), which are arranged at sites corresponding to hydrophobic regions (Fig. 4A). H1–36 are predicted to form 9 THUs, each containing 4 contiguous transmembrane helices. Furthermore, a large intracellular region and a large extracellular region were found between H28 (THU7) and H29 (THU8), and between H37 and H38, respectively. The protein structure of PxanPiezo was constructed using homology modeling, which presents the tertiary structures of PxanPiezo from THU4 to the C-terminus. In the PxanPiezo monomer, the THUs form a blade-like structure. H37 and H38, along with several semi-transmembrane helices, form a central pore region. The extracellular region between H37 and H38 forms a central cap structure (Fig. 4B). A long, bent helix in the intracellular region between THU7 and THU8 forms a beam-like structure on the intracellular side that bridges the blade and the central regions. The top view of the PxanPiezo trimer is characterized by a 3-bladed, propeller-shaped structure, and the side view resembles a funnel covered with a central cap (Fig. 4C and D).

### Characterization of NOMPC in *P. xanthodes*

**Fig. 5.**
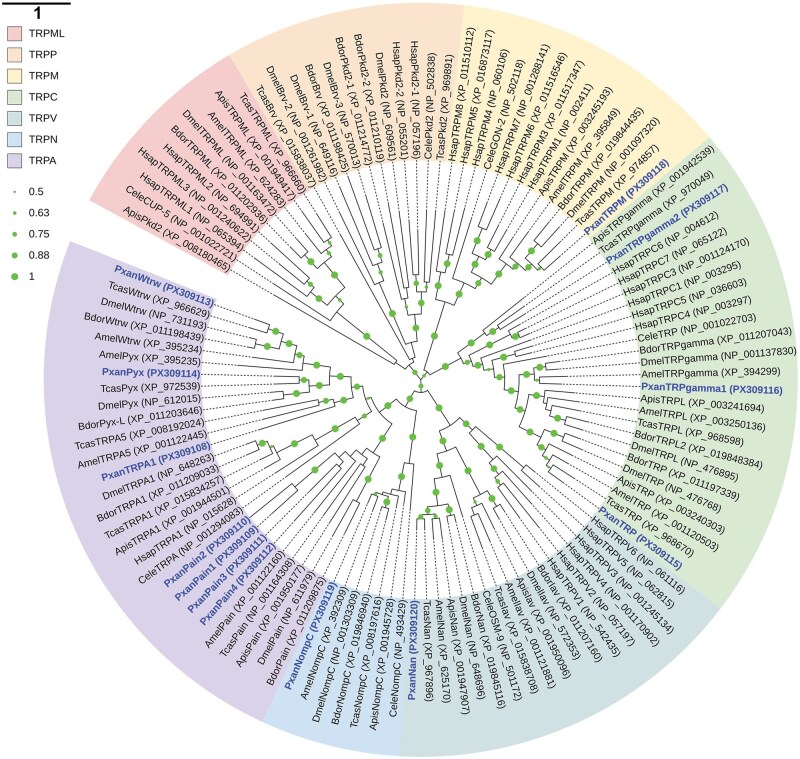
Phylogenetic analysis of PxanTRPs. Insect TRPs are assigned to 7 subfamilies: TRPA, TRPN, TRPV, TRPC, TRPM, TRPML, and TRPP. The GenBank accession numbers are given in the brackets after the TRP names. Bootstrap values below 50% are not shown.

Thirteen TRPs were identified in *P. xanthodes* (Table 1). The ORFs range from 2,430 to 5,238 bp and encodes proteins with 809 to 1,745 amino acids. Phylogenetic analysis showed that animal TRPs are grouped into 7 subfamilies (Fig. 5). PxanTRPs are distributed in the following subfamilies: TRPAs (*PxanTRPA1*, *PxanPain1*, *PxanPain2*, *PxanPain3*, *PxanPain4*, *PxanWtrw*, and *PxanPyx*), TRPCs (*PxanTRP*, *PxanTRPgamma1*, and *PxanTRPgamma2*), TRPM (*PxanTRPM*), TRPN (*PxanNompC*), and TRPV (*PxanNan*). PxanNompC shares a close relationship with NompCs in other insects. Its identities with NompCs in *Chrysoperla carnea*, *T. castaneum*, and *D. melanogaster* are 82.10%, 78.40% and 75.22%, respectively. In contrast, its identity with other PxanTRPs is relatively low, ranging from 21.28% to 31.29%.

**Fig. 6.**
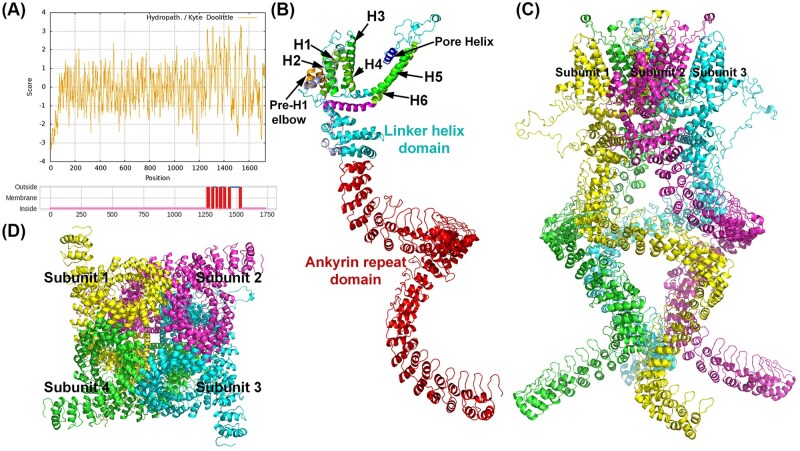
Structural analysis of PxanNompC. A) Predicted hydrophobic regions and transmembrane helices in PxanNompC. Positive score indicates hydrophobicity. Six transmembrane helices (H1–6) and 2 hydrophobic membrane-embedded helices before H1 are located on the sites corresponding to hydrophobic regions. B) Ribbon diagram of the structure of PxanNompC. A spring-shaped ankyrin repeat domain, a linker helix domain, and a transmembrane domain mainly formed by H1–6, the membrane-embedded helices (Pre-H1 elbow) and a pore helix were found in PxanNompC. C) Side view of PxanNompC tetramer. D) Bottom view of PxanNompC tetramer.

PxanNompC is a transmembrane protein comprising a hydrophobic region, a large intracellular N-terminal region and a shorter C-terminal region. The hydrophobic region contains 2 membrane-embedded hydrophobic helices and 6 transmembrane helices (H1–H6) (Fig. 6A). Conserved TRP domain “trp” (TIGR00870) was found covering the transmembrane region. Conserved ankyrin repeat domains including “ANKYR” (COG0666), “Ank_2” (pfam12796), and “ANK” (smart00248) were found in the N-terminal region. Homology modelling showed that the ankyrin repeat domain appears to act as a helical spring constructed by 29 ankyrin repeats, each of which is a conserved helix–loop–helix structure (Fig. 6B). The transmembrane helices in the hydrophobic region form 2 helix bouquets, which are composed of H1–4 and H5–6, respectively. Two hydrophobic helices near H1 form an elbow-like structure. A reentrant pore helix is located on the site between H5 and H6. In the homotetramer, the N-terminal ankyrin repeat domains of each subunit are organized into a cytoplasmic domain of quadruple spring structure (Fig. 6C and D). The transmembrane helices H5 and H6, and the pore helix are involved in forming a central ion channel.

### Expression Profile of PxanPiezo and PxanTRPs

**Fig. 7.**
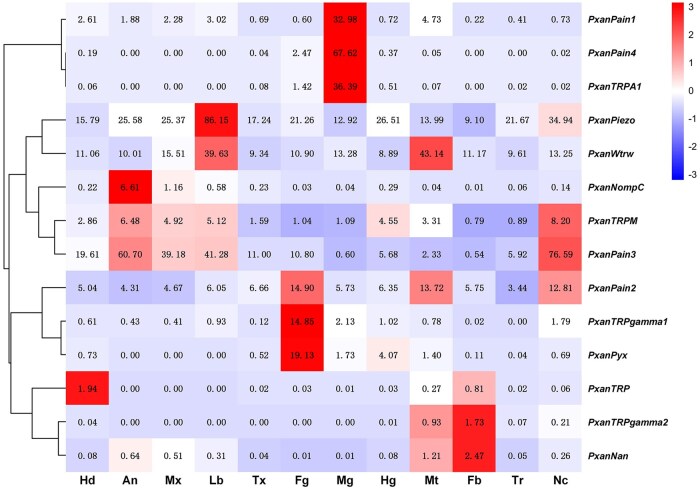
Heat map of PsonPiezo and PsonTRP genes. Cluster analyses were based on FPKM. Each column represents a sample and each row represents a gene. Relative expression levels of genes in different samples are indicated by a color scale ranging from red to blue. Red boxes represent highly expressed genes. Blue boxes represent lowly expressed genes. FPKM values are shown in the boxes. Hd, Head; An, antennae; Mx, Maxillae; Lb, Labium; Tx, Thorax; Fg, Foregut; Mg, Midgut; Hg, Hindgut; Mt, Malpighian tubules; Fb, Fat body; Tr, Tracheae; Nc, Nerve cord.

The expression profiles of PxanPiezo and PxanTRPs in the larvae were analyzed based on FPKM values. *PxanPiezo*, *PxanWtrw*, and *PxanPain2* are widely expressed throughout the head, thorax, and surface tissues, with FPKM values greater than 1 (Fig. 7). *PxanPain3* is also widely expressed across different tissues, with the highest expression in the nerve cord. However, its expression levels in the midgut and fatbody are lower than in other tissues (FPKM < 1). *PxanPain1*, *PxanPain4*, and *PxanTRPA1* are specifically expressed in the midgut. *PxanTRPgamma1* and *PxanPyx* are mainly detected in the foregut. *PxanNompC* is detected in the antennae and maxillae, and the FPKM values are 6.61 and 1.16, respectively, which are much lower than that of *PxanPiezo*. *PxanTRP*, *PxanTRPgamma2*, and *PxanNan* are only expressed in a few tissues with FPKM values higher than 1 (Fig. 7).

### Tissue Distribution of PxanPiezo and PxanNompC in the Body Surface Structures

**Fig. 8.**
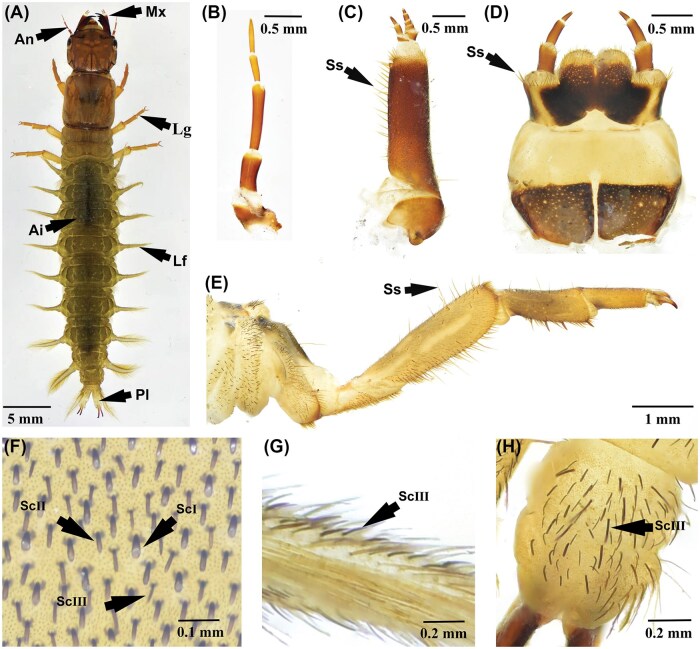
Body surface structures of *P. xanthodes* larvae. A) Dorsal view of the larva; B) Antenna; C) Maxilla; D) Labium; E) Thorax leg; F) Abdominal integument. The club-shaped setae (Sc) are categorized into three types: ScI is straight and gradually widens distally; ScII is straight and lacks a swollen tip; ScIII is slightly curved and also lacks a swollen tip; G) Lateral filament; H) Anal prolegs; An, antennae; Mx, maxillae; Lb, labium; Lg, legs; Lf, lateral filaments; Pl, prolegs; Ai, abdominal integument; Ss, spine-like seta.

**Fig. 9.**
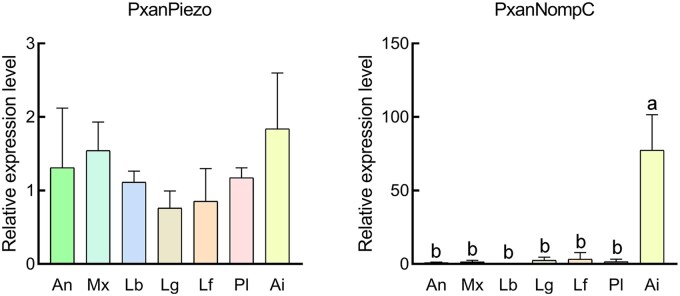
Expression of PxanPiezo and PxanNompC in body surface structures of larvae. An, antennae; Mx, maxillae; Lb, labium; Lg, legs; Lf, lateral filaments; Pl, prolegs; Ai, abdominal integument. Error bars show the standard errors of the means of 3 biological replicates. Significant differences are marked with letters (*P* < 0.05, 1-way ANOVA). All values are mean ± SE.

The whole body of *P. xanthods* larva is covered with integument (Fig. 8). Spine-like setae are distributed on the maxillae and labium of the mouthparts, and on the coxa, trochanter, femur, and tibia of the thoracic legs (Fig. 8C–E). Club-shaped setae are distributed on the abdominal surface, lateral filaments, and anal prolegs (Fig. 8F–H). Notably, the dorsal surface of the abdomen is densely covered with club-shaped setae. RT-qPCR revealed that *PxanPiezo* is widely expressed in the antennae, maxillae, labium, legs, lateral filaments, anal prolegs, and abdominal integument with no significant difference observed between these tissues (Fig. 9). *PxanNompC* exhibits tissue-specific expression among the body surface structures and is highly expressed in the abdominal integument (Fig. 9).

## Discussion

Insects perceive the surrounding mechanical stimuli via mechanosensory organs that express mechanotransduction channels for mechanoelectrical transduction (Tuthill and Wilson 2016). In aquatic insects, the ability to perceive flowing water and other mechanical stimuli is essential for their survival in a freshwater environment (Piersanti et al. 2024). In this study, we provided a transcriptome analysis of the maxillae, labium, and antennae of *P. xanthodes* larvae, and identified for the first time presenting genes encoding Piezo and NompC in this aquatic insect. PxanPiezo is a large transmembrane protein containing 38 transmembrane helices. Its trimers may play a role in mechanosensation by transducing membrane stretch into a signaling current. PxanNompC is a transmembrane protein with a large intracellular domain consisting of ankyrin repeats, conserved structures involved in protein–protein interactions, and may be involved in the signal transduction in response to cellular tension. FPKM analysis showed that *PxanPiezo* and *PxanNompC* differ in expression profiles. *PxanPiezo* is ubiquitous in the sensory organs (antennae), mouthparts (maxillae and labium), nerve tissues (head and nerve cord), motor tissue (thorax), digestive organs (foregut, midgut and hindgut), excretory organ (Malpighian tubules), energy storage tissue (fatbody), and the respiratory tissue (tracheae); *PxanNompC* is found at low FPKM values in the antennae and maxillae. RT-qPCR indicated that PxanPiezo is widely expressed in the body surface tissues of *P. xanthodes* larvae. *PxanNompC* exhibits tissue-specific expression among the body surface structures, being enriched in the abdominal integument. These results suggest that PxanPiezo and PxanNompC are candidate mechanoelectrical transduction channels. Tissues expressing the *PxanPiezo* and *PxanNompC* genes may play an important role in perceiving the internal and environmental mechanical stimuli.

Animal Piezos are transmembrane channels that are highly associated with cytomembrane (Xiao 2024). Structural analysis of Piezo1 and Piezo2 in *M. musculus* revealed that animal Piezos exist as trimers on the membrane, forming an ion channel that is controlled by cell deformation located at the center of the trimer (Zhao et al. 2018). A Piezo trimer viewed from above resembles a propeller with 3 paddles. These propeller-shaped structures interact with the cytomembrane to form a funnel-shaped structure through which changes in membrane curvature generate a gating force for the central ion channel (Wang et al. 2019). In this study, PxanPiezo is a large transmembrane protein comprising 38 transmembrane helices, which correspond to those found in MmPiezos in *M. musculus* (Saotome et al. 2018, Wang et al. 2019). The first 36 transmembrane helices in MmPiezo form 9 THUs, which construct a propeller-like structure within the trimers and play a vital role in converting membrane stretch into the gating force (Saotome et al. 2018, Zhao et al. 2018, Kefauver et al. 2020, Fang et al. 2021). A long intracellular beam located between THU7 and THU8 supports the transcriptome skeleton and physically bridges the distal THUs to the central ion-conducting pore (Fang et al. 2021). Membrane stretch leads to flattening of the distal blade and levering of the beam, which in turn gates the intracellular lateral plug gate of the pore (Wang et al. 2019, Fang et al. 2021, Yang et al. 2022). In *P. xanthodes*, PxanPiezo also has a beam helix that bridges the blade-like structure to the central pore region. Therefore, the structural similarity between PxanPiezo and mechanosensitive Piezos in other animals suggests that PxanPiezo has the structural basis for mechanoelectrical transduction and may play a role in the mechanosensory system of *P. xanthodes*.

NompC belongs to the TRP family which is divided into 7 subfamilies (Himmel and Cox 2020). Each subfamily contains a conserved transmembrane domain with 6 transmembrane helices but varies in the structural features of the intracellular domain (Zhang et al. 2013). The NompC homotetramer functions as a tether-based mechano-gated ion channel involved in mechanotransduction for touch sensation and hearing (Wang et al. 2022). Structurally, the homotetramer contains 29 ankyrin repeats per subunit, organized into a quadruple helical spring architecture (Argudo et al. 2019). These ankyrin repeats are associated with microtubules in the cytoskeleton and are thought to act as a tether that conveys force to the channel (Liang et al. 2013, Zhang et al. 2023). In *P. xanthodes*, PxanNompC is a 6-transmembrane protein with a large intracellular region consisting of ankyrin repeats, and it may function as a homotetramer channel with a central pore. It shares high sequence identity and similar structural features with DmelNompC in *D. melanogaster*, which is involved in touch sensation, proprioception, and hearing (Jin et al. 2017). Evidence in the literature showed that DmelNompC mediates mechanosensation by converting mechanical stimuli into electrical signals (Argudo et al. 2019). The ankyrin repeats in DmelNompC are tethered to the microtubules in the cytoskeleton, conveying mechanical force to gate the central pore (Zhang et al. 2023). Therefore, we suggest that PxanNompC, which forms a homotetramer comprising a transmembrane domain and an ankyrin repeat domain, may play a role in mechanosensation by converting the traction of the cytoskeleton into ionic currents.

In *P. xanthodes*, PxanPiezo and PxanNompC demonstrate distinct expression patterns in larval tissues. Expression analysis revealed that PxanPiezo is widely expressed in various tissues, including sensory organs, mouthparts, nerve tissues, motor tissues, digestive organs, excretory organs, energy storage tissues, and respiratory structures, as well as a variety of body surface tissues, suggesting a potential role in sensing environmental stimuli and proprioception, while PxanNompC is primarily expressed in the abdominal integument and is also found in the antennae and maxillae. The distinct tissue distribution patterns suggest that *PxanPiezo* and *PxanNompC* may have functional differences. In *D. melanogaster*, DmelPiezo exhibits broad and functionally significant expression across multiple tissues, including sensory organs, the nervous system, the digestive tract, excretory tissues, and respiratory structures, with particularly high levels observed in the labium (Suslak et al. 2015, He et al. 2018, Puri et al. 2025). Functionally, DmelPiezo is essential for nociceptive neurons by sensing noxious mechanical stimuli (Kim et al. 2012). It evaluates substrate stiffness during egg-laying, coordinates feeding and swallowing rhythms via pharyngeal sensors, conveys gut distension signals to control meal size, and mediates proprioception for normal locomotion (Zhang et al. 2015, Hu et al. 2019, Min et al. 2021, Oh et al. 2021, Yamanouchi et al. 2023). These functions highlight DmelPiezo as a multifunctional channel that is vital for sensory processes, motor processes, homeostatic regulation, and stem cell differentiation (Kim et al. 2012, He et al. 2018, Schulz et al. 2024). Furthermore, Piezo and NompC are also co-expressed in certain tissues, where they play complementary roles in mechanosensory behaviors. In the neurons around *Drosophila* larval cibarium, DmelPiezo and DmelNompC are both involved in controlling the food swallowing process. DmelNompC is proposed to have a role in initiation, while DmelPiezo controls the driving strength of swallowing (Qin et al. 2024). Therefore, we suggest that PxanPiezo may function as a house-keeping mechanoelectrical transduction channel that responds to general mechanical stimuli, both of internal or external origin.

Unlike the ubiquitous PxanPizo, the expression of PxanNompC is highly concentrated in the abdominal integument, which is covered in a large number of club-shaped setae. The setae on the insect integument often act as sensilla, functioning as crucial intermediaries for transmitting external stimuli to the internal sensory neurons, as the integument is tough and rigid, separating the body from the external environment (Berni 2024). In the freshwater environment, aquatic insects sense fluid flow using highly sensitive mechanosensory sensilla, which are often abundant on the surface of body and appendages (Casas and Dangles 2010). At the base of these sensilla, there are dendritic tips of sensory neurons that express sensory genes and convert external stimuli into neuroelectric signals (Xiao 2024). Evidence in the literature has indicated that insect NompC is widely found at the tips of mechanosensory cilia in various sensory neurons, including chordotonal organs and sensilla (Sidi et al. 2003, Lee et al. 2010, Sun et al. 2019,  Zhang et al. 2020). *P. xanthodes* larva are aquatic predators in the benthic invertebrate communities with integument directly contacted with the external water flow (Yang et al. 2025). The setae and PxanNompC in their integument may function as the structural and molecular bases, respectively, for perceiving mechanical cues in many behavioral and ecological contexts. In *Drosophila*, *DmelNompC* is required for both sound detection and mechanical amplification in auditory receptors (Effertz et al. 2011, Li and Yan 2021). It also regulates the locomotion of *Drosophila*; its knockdown leads to severe movement defects (Cheng et al. 2010, Zanini et al. 2018). Furthermore, insect NompC contributes to processes such as the detection of food texture, the coordination of swallowing, and even the sensing of noxious cold (Turner et al. 2016, Qin et al. 2024). Knockout or knockdown of NompC results in dramatically reduced survival rates, morphological deformities, and lethality, highlighting its indispensable role in insect survival (Tsubouchi et al. 2012, Su et al. 2025b). Therefore, we suggest that the abdominal integument of *P. xanthodes* larvae carrying density setae plays an important role in perceiving external stimuli, such as water flow. PxanNompC is highly expressed in the integument and may be functionally involved in transducing external stimulus signals from the freshwater environment.

In conclusion, we identified 2 mechanoelectrical transduction channels (*PxanPiezo* and *PxanNompC*), in the predatory aquatic insect *P. xanthodes*. *PxanPiezo* is ubiquitously expressed in the internal and surface tissues of *P. xanthodes* larvae; whereas *PxanNompC* shows a very specific expression pattern, being highly expressed in the abdominal integument, which is densely covered with club-like setae. These results suggest that PxanPiezo and PxanNompC may differ in their mechanoelectrical transduction mechanisms and likely define complementary mechanosensory pathways: PxanPiezo functions as a general mechanoreceptor that detects both internal and environmental stimuli, whereas PxanNompC appears to be restricted to specific tissues and is likely specialized in perceiving particular types of environmental mechanical stimuli. However, a direct functional validation is currently lacking. Future investigations, for instance through behavioral assays or gene knockdown experiments, are still needed to define the roles of PxanPiezo and PxanNompC in mechanosensory pathways.

## Supplementary Material

ieaf102_Supplementary_Data
