# Supplementary material for: Identification of candidate mechanosensory transduction channels in the aquatic insect Protohermes xanthodes (Megaloptera: Corydalidae) by transcriptome analysis
Source: J Insect Sci. 2025 Nov 22;25(6):ieaf102. doi: 10.1093/jisesa/ieaf102 (PMC12640122; doi:10.1093/jisesa/ieaf102)
Supplement: ieaf102_Supplementary_Data [file ieaf102_supplementary_data.zip › 6 Supplementary File.doc]

**Table S1. Primers used for RT-qPCR**

| Primers | Primer Sequence (5’-3’) |
| --- | --- |
| *PxanPiezoF* | CTAGCGTTGAGTAGAGGCCG |
| *PxanPiezoR* | TTCACCTTCGAGCGCTACAG |
| *PxanNompCF* | ATTCTGCCGGCGTTCAAGTA |
| *PxanNompCR* | CAATGGCGTCCAACCGTTTT |
| *α-tubulinF* | TGGCAGGTTCAAAACAAGCG |
| *α-tubulinR* | TCGATGGTGCCCTCAATGTT |

**Table S2. Statistics of cDNA libraries**

| Sample | Raw reads | Clean reads | Clean bases | Error rate | Q20 | Q30 | GC percentage |
| --- | --- | --- | --- | --- | --- | --- | --- |
| Antenna 1 | 38356191 | 37002786 | 11.10 | 0.01 | 98.92 | 96.74 | 40.52 |
| Antenna 2 | 34468068 | 33395219 | 10.02 | 0.01 | 98.91 | 96.72 | 42.21 |
| Antenna 3 | 39090171 | 37526364 | 11.26 | 0.01 | 98.99 | 97.01 | 41.81 |
| Maxillae 1 | 38651616 | 37149302 | 11.14 | 0.01 | 99.00 | 97.01 | 40.59 |
| Maxillae 2 | 40393064 | 38145330 | 11.44 | 0.01 | 98.96 | 96.89 | 40.72 |
| Maxillae 3 | 34485783 | 32803045 | 9.84 | 0.01 | 98.95 | 96.85 | 40.23 |
| Labium 1 | 38769081 | 37778735 | 11.33 | 0.01 | 99.02 | 97.01 | 40.48 |
| Labium 2 | 37974913 | 36631569 | 10.99 | 0.01 | 98.99 | 96.98 | 41.7 |
| Labium 3 | 39694030 | 38511839 | 11.55 | 0.01 | 99.05 | 97.16 | 40.52 |

**Table S3. Statistics of Trinity assembly**

| Type | Min length | Mean length | Median length | Max length | N50 | N90 | Total nucleotides | Number |
| --- | --- | --- | --- | --- | --- | --- | --- | --- |
| Transcript | 301 | 1175 | 609 | 53198 | 2029 | 446 | 207923874 | 177000 |
| Unigene | 301 | 890 | 530 | 53198 | 1172 | 396 | 90097568 | 101286 |

**Table S4. Statistics of gene annotation**

| Data base | Number of Unigenes | Percentage (%) |
| --- | --- | --- |
| Annotated in NR | 49490 | 48.86 |
| Annotated in NT | 43226 | 42.67 |
| Annotated in KO | 30125 | 29.74 |
| Annotated in SwissProt | 37900 | 37.41 |
| Annotated in PFAM | 35683 | 35.22 |
| Annotated in GO | 35683 | 35.22 |
| Annotated in KOG | 23609 | 23.3 |
| Annotated in all Databases | 10321 | 10.18 |
| Annotated in at least one Database | 67921 | 67.05 |
| Total Unigenes | 101286 | 100 |
